# Psychological outcomes amongst family medicine healthcare professionals during COVID-19 outbreak: A cross-sectional study in Croatia

**DOI:** 10.1080/13814788.2021.1954154

**Published:** 2021-07-30

**Authors:** Sunčana Vlah Tomičević, Valerija Bralić Lang

**Affiliations:** a Health Centre Zagreb – East, Zagreb, Croatia; b Private Family Physician Office, Department of Family Medicine, Zagreb University School of Medicine, School of Public Health ‘Andrija Štampar’, Zagreb, Croatia

**Keywords:** Family medicine, stress, anxiety, depression, PTSD

## Abstract

**Background:**

Healthcare professionals (HCPs) in family medicine (FM) in Croatia work in a demanding environment caused by the SARS-CoV-2 pandemic. Besides particular circumstances in healthcare, an unknown virus, social distancing, and homeschooling, the capital was hit with the earthquake during the lockdown.

**Objectives:**

To assess the prevalence of stress, anxiety, depression, posttraumatic stress disorder (PTSD) and the influence of demographic characteristics, professional differences, medical history, and specific stressors on the psychological outcomes.

**Methods:**

A cross-sectional study with the online questionnaire containing the Depression, Anxiety and Stress Scale (DASS-21) and the Impact of Event Scale-Revised (IES-R) was conducted from 1st to 15 May 2020 in FM.

**Results:**

HCPs (534, 35% response rate), predominantly female (84.5%), participated in the research. High prevalence of stress (30.9%), anxiety (33.1%), depression (30.7%), and PTSD (33.0%) were found. Female participants had higher results in the anxiety subscale of DASS-21 and IES-R scores. Pre-existing conditions were associated with higher levels of stress, anxiety, depression, and PTSD. The IES-R score for PTSD showed borderline correlation (*p* = 0.053) with working in regions with the highest incidence of COVID-19. Having schoolchildren made a difference on a stress subscale in DASS-21 (*p* < 0.043), but the earthquake did not have an impact.

**Conclusion:**

Family physicians and nurses in FM in Croatia are under a great mental load during the COVID-19 outbreak. Results suggest that HCPs of the female sex, with pre-existing chronic conditions, work in regions with a high incidence of SARS-CoV-2 or have schoolchildren at greater risk of the poor psychological outcome.

KEY MESSAGES

- There is a high prevalence of stress, anxiety, depression, and PTSD among HCPs working in FM in Croatia during the SARS-CoV-2 outbreak
- Low mental preparedness, social isolation, extreme workloads, and poor communication with the government had psychological effects

## Introduction

High psychological stress in everyday life leads to mental disorders in all healthcare professionals (HCPs), especially nurses and junior doctors [1,2]. Unexpected events, like the global spread of novel coronavirus, combined with social distancing, leave a serious toll on people’s mental health [3].

In March 2020, the Croatian Minister of Health declared the danger of the SARS-CoV-2 epidemic, and only a week later, WHO announced the COVID-19 outbreak a pandemic. Protective measures like visitation ban for retirement homes, wearing face masks, social distancing, and in the end, a total lockdown was imposed in everyday life. A change in the functioning of the healthcare system was made for daily practice in family medicine (FM) by focussing on infectious diseases, communicating with patients *via* phone or e-mail rather than in person, using some unusual personal protective equipment (PPE) for FM, and working overtime in new specialised practices for patients’ triage. In the first weeks of the pandemic, various work instructions arrived daily and were often illogical to apply in primary care. To date, there are no primary health care representatives in Croatia’s national headquarters for the fight against COVID-19. Hospital HCPs started to attend only emergency and urgent interventions working in 14 d shifts. Fear of the unknown among HCPs and the inability to protect the patients and their loved ones were present. With ageing, the incidence of chronic non-communicable diseases rises [4]. Croatia’s population is predominantly old, with on average three generations living in the same household. So, the chance for HCPs to have comorbidities or live with people with multimorbidity is high, placing all of them in the risk group for a more severe case of COVID-19 [5]. With only online school classes, HCPs who have children needed to ensure a safe home environment, equipment for the classrooms, and help with homework and studying. During the SARS-CoV-2 lockdown on 22nd March 2020, Croatia’s capital Zagreb was hit with the strongest earthquake in the last 140 years that resulted in 1 death, injured 27 people, damaged more than 25,000 buildings, including some hospitals, and brought additional anxiety, and a more stressful everyday life [6].

This study aimed to assess the prevalence of stress, anxiety, depression, and PTSD among HCPs working in FM in Croatia during the SARS-CoV-2 outbreak and their association with demographic characteristics, professional differences, medical history, and specific stressors among the participants.

## Methods

### Study design and recruitment

A cross-sectional study was conducted among FM HCPs employed in primary care in Croatia. From the most recent Croatian Health Statistics Yearbook 2018, 2298 family physician (FP) teams included a doctor and a single nurse [7]. The participants were recruited *via* an e-mail notification that contained a link to the web version of the questionnaire set up *via* the SurveyMonkey^®^ platform (SurveyMonkey Inc., Palo Alto, CA). The software did not collect respondents’ IP addresses and was completely anonymous and voluntary. The survey was conducted from 1 May to 15 May 2020. The two largest professional societies of FPs distributed the online version of the questionnaire among their members with one reminder. Since some doctors have more than one e-mail address, are members of two or more societies or are already retired, we could not calculate the actual response rate but only a rough estimation of 35%.

### Questionnaire

The study questionnaire was approved by the Medical Ethics Committee of the Health Center Zagreb – Centar and was written in Croatian. It comprised demographic characteristics (age, sex, marital status, place of work), professional differences (doctor or nurse, years of work experience), medical history (data on comorbidity), specific stressors (having schoolchildren, earthquake, high COVID-19 incidence in region of work), the Depression, Anxiety and Stress Scale (DASS-21), and the Impact of Events Scale-Revised (IES-R) instruments.

DASS-21 is a self-reported 21-item system that provides independent measures of stress, anxiety, and depression with recommended severity thresholds. Cut-off scores of >14 for stress, >7 for anxiety, and >9 for depression, obtained by multiplying a final score number with two, represent a positive screening for stress, anxiety, and depression, respectively and were used to calculate the prevalence of each. The DASS-21 stress subscale score was divided into ‘mild’ (15–18), ‘moderate’ (19–25), ‘severe’ (26–33), and ‘extremely severe’ stress (34–42). Anxiety subscale score was assessed as ‘mild’ (8–9), ‘moderate’ (10–14), ‘severe’ (15–19), and ‘extremely severe’ (20–42). On the depression subscales, scores of 10–13 were deemed as ‘mild,’ 14–20 as ‘moderate,’ 21–27 as ‘severe,’ and 28–42 as ‘extremely severe’ depression [8].

The psychological distress of the outbreak was assessed using the IES-R, a validated 22-item self-report that measures the subjective distress caused by traumatic events [9]. It has three subscales (Intrusion, Avoidance, and Hyperarousal) closely affiliated with posttraumatic stress disorder (PTSD) symptoms. Participants were asked to rate the level of distress for each component during the previous month of their interview. The total IES-R score was graded for severity from normal (0–23), mild (24–32), moderate (33–36), and severe (>37) psychological impact with a cut-off score of ≥24 to define PTSD of clinical concern.

Both DASS-21 and IES-R have been validated for use in Croatia with a high Cronbach’s α reliability coefficient for the DASS-21 questionnaire (0.90, 0.82, 0.88) and IES-R (0.79 to 0.94) depending on the subscale (10–12).

### Study outcome

The primary outcome was the prevalence of stress, anxiety, depression, and PTSD among Croatian doctors and nurses in FM. As secondary outcomes, we looked at the association between the prevalence of stress, anxiety, depression, PTSD, and median DASS-21 and IES-R scores and demographic characteristics, professional differences, medical history, and specific stressors (having schoolchildren, earthquake, high COVID-19 incidence in region of work).

### Statistical analysis

The distribution of quantitative data was tested by the Kolmogorov–Smirnov test. Quantitative data were presented as median (min-max). Categorical data were presented as absolute and relative frequencies. Depending on the data, we performed a Mann-Whitney U test (two groups), Kruskal–Wallis test (three and more groups), or *χ*^2^-test to compare the occurrence of individual conditions. The level of significance (*p*) was 2-tailed and *p* values < 0.05 were considered statistically significant. Multivariate logistic regression analysis was performed to determine the extent to which the different variables independently predict the probability of stress, anxiety, depression, and PTSD. The predictors included in the regression analyses were age, sex, marital status, professional differences, presence of chronic diseases, having schoolchildren, and working in areas hit with an earthquake and high incidence of COVID-19 (Zagreb). An error threshold of *α* = 0.05 was used in the interpretation of the results. All data analyses were performed using SPSS ver. 23.0, Armonk, NY; IBM Corp.

## Results

A total of 534 HCPs filled the questionnaire, representing 19.5% of all doctors and 3.7% of all nurses currently working with FPs in Croatia. Baseline characteristics and their distribution are shown in Table 1. Compared to the total population of FP’s and nurses in Croatia concerning sex, we found no differences (*p* = 0.059, *p* = 0.268), neither when comparing the age of the nurses (*p* = 0.126). In the broader Zagreb region, more doctors who were younger than 45 filled in the questionnaire (*p* < 0.001), so our sample for that region has significantly younger FPs (*p* < 0.001). The distribution by age categories for all other counties was representative. More than a quarter (26.6%) of all participants had children in elementary or high school and a total of 338 (63.3%) of all participants had at least one non-communicable chronic disease.

**Table 1.** Baseline characteristics of study participants.

Characteristics	Total *N* (%)	Doctor *N* (%)	Nurse *N* (%)
Number of participants	534 (100)	448 (100)	86 (100)
Sex			
Female	451 (84.5)	366 (81.7)	85 (98.8)
Male	83 (15.5)	82 (18.3)	1 (1.2)
Age			
18–44	209 (39.1)	164 (36.6)	45 (52.3)
≥45	325 (60.9)	284 (63.4)	41 (47.7)
Work experience (years)			
0–10	153 (28.7)	126 (28.1)	27 (31.3)
11–30	223 (41.8)	188 (42.0)	35 (40.7)
>30	158 (29.6)	134 (29.9)	24 (27.9)
Marital status			
With partner	385 (72.1)	332 (74.1)	53 (61.6)
Without partner	149 (27.9)	116 (25.9)	33 (38.4)
Medical history			
Arterial hypertension	99 (18.5)	90 (20.1)	9 (10.5)
Diabetes mellitus	18 (3.4)	16 (3.6)	2 (2.3)
Ischemic heart disease	4 (0.7)	2 (0.4)	2 (2.3)
Stroke	2 (0.4)	2 (0.4)	0 (0)
Hyperlipidemia	63 (11.8)	59 (13.2)	4 (4.7)
Migraine	46 (8.6)	29 (6.5)	17 (19.8)
Gastritis/GERB	114 (21.3)	96 (21.4)	18 (20.9)
Psychiatric illness	18 (3.4)	16 (3.6)	2 (2.3)
Cancer	24 (4.5)	21 (4.7)	3 (3.5)
Skin diseases	29 (5.4)	23 (5.1)	6 (7.0)
Asthma	22 (4.1)	17 (3.8)	5 (5.8)
Smoker	79 (14.8)	58 (12.9)	21 (24.4)
No pre-existing condition	194 (36.3)	166 (37.1)	28 (32.6)

The prevalence of stress was 30.9%, anxiety 33.1%, depression 30.7%, and clinically relevant score for PTSD was present in 33.0% of the participants with median results for subscale Intrusion 5 (0–31), Avoidance 6.5 (0–29), and Hyperarousal 4 (0–23) (Table 2). The sex difference was found in the anxiety subscale of DASS-21 and also in IES-R scores (*p* < 0.038 and *p* < 0.000). In regions with the highest incidence of COVID-19 (Zagreb and Split-Dalmatia’s County), the HCP’s IES-R score for PTSD showed borderline correlation (*p* = 0.053). Having schoolchildren made a difference on a stress subscale in DASS-21 (*p* = 0.043). A third of participants (33.5%) were from Zagreb, a region affected by the earthquake, but that kind of stressor showed no impact on their DASS-21 or IES-R score (all *p*’*s* > 0.05).

**Table 2.** Prevalence of different levels of stress, anxiety, depression with median DASS-21 and IES-R scores*.

Outcome	Categories					
DASS-21	*N*ormal *N* (%)	Mild *N* (%)	Moderate *N* (%)	Severe *N* (%)	Extremely severe *N* (%)	Median (min-max) scores
DASS-21 stress	368	52	68	32	14	6
	(68.9)	(9.7)	(12.7)	(5.9)	(2.6)	(0–21)
DASS-21 anxiety	357	69	54	24	30	2
	(66.85)	(12.9)	(10.1)	(4.5)	(5.6)	(0–19)
DASS-21 depression	369	64	68	19	14	2
	(69.10)	(11.9)	(12.7)	(3.5)	(2.6)	(0–21)
IES-R	Normal	Clinical concern	Probable	Expressed	/	Median (min-max) scores
	357	71	32	74	/	15
	(66.8)	(13.3)	(5.9)	(13.8)		(0–82)

*DASS-21 cut-off scores of >14 for stress: mild (15–18), moderate (19–25), severe (26–33), extremely severe (34–42); >7 for anxiety: mild (8–9), moderate (10–14), severe (15–19), extremely severe (20–42); and >9 for depression: mild (10–13), moderate (14–20), severe (21–27), extremely severe (28–42).

IES-R cut-off scores for PTSD ≥24: for mild (24–32), moderate (33–36), and severe (>37).

After further adjustment for age, sex, marital status, occupation, years of work experience, presence of chronic disease, having schoolchildren, and workplace, age ≥45 was positively associated with anxiety (*p* = 0.042) and female sex with IES-R (*p* = 0.011) (Table 3).

**Table 3.** Multivariable logistic regression model for DASS-21 and IES-R scores.

	Stress		Anxiety		Depression		IES-R	
Variable	OR		OR		OR		OR	*p*
	(95%CI)	*p*	(95%CI)	*p*	(95%CI)	*p*	(95%CI)	
Age (years):		0.099		0.042		0.075		0.791
<45	0.502		0.565		0.522		0.948	
>45	(0.221–1.139)		(0.326–0.98)		(0.255–1.068)		(0.637–1.41)	
Sex:		0.580		0.880		0.179		0.011
Men	0.77		1.057		2.109		2.121	
Women	(0.305–1.943		(0.515–2.169)		(0.711–6.259)		(1.186–3.792)	
Marital status:		0.301		0.865		0.857		0.195
With partner	1.221		1.024		0.968		0.868	
Without partner	(0.837–1.781)		(0.782–1.34)		(0.68–1.378)		(0.701–1.075)	
Profession:		0.117		0.732		0.210		0.394
Doctor	3.04		0.882		1.95		1.281	
Nurses	(0.757–12.207)		(0.431–1.807)		(0.687–5.537)		(0.725–2.266)	
Work experience: (years)		0.780		0.256		0.269		0.995
<20	1.107		1.325		1.42		0.999	
>20	(0.544–2.251)		(0.815–2.155)		(0.762–2.645)		(0.702–1.421)	
Chronic disease:		0.000		<0.001		0.002		<0.001
No	5.394		2.656		3.019		2.882	
Yes	(2.345–12.407)		(1.567–4.501)		(1.525–5.977)		(1.94–4.283)	
Having schoolchildren:		0.149		0.620		0.282		0.075
No	1.852		1.158		0.624		1.481	
Yes	(0.802–4.278)		(0.649–2.064)		(0.264–1.473)		(0.961–2.284)	
Workplace:		0.718		0.895		0.413		0.351
Other	0.862		0.963		1.332		1.219	
Zagreb	(0.384–1.932)		(0.556–1.671)		(0.67–2.649)		(0.804–1.847)	

OR – odds ratio, OR </>1: presence of variable reduces/raises the odds of the event, CI – confidence interval, IES-R – impact of event scale-revised, *p – p*-value.

## Discussion

### Main findings

Our research showed a high prevalence of stress (30.9%), anxiety (33.1%), depression (30.7%), and PTSD (33.0%) among HCPs in FM during the COVID-19 outbreak in Croatia. Anxiety and PTSD scores were higher in women, and HCPs with at least one chronic disease were more likely to have higher stress levels, anxiety, depression, and symptoms that indicate PTSD. The older age was shown to be a risk factor for anxiety symptoms solely. Another relevant stressor was having schoolchildren while living in high incidence regions of COVID-19 had a borderline effect on IES-R score. Unexpectedly, we did not find an impact on the HCPs IES-R score in the earthquake-hit area.

### Strengths and limitations

This study’s strengths include the high number of respondents, the use of validated measurement tools, and the specific circumstance of the concomitant earthquake in Croatia with the pandemic, which was not found in other studies on the psychological influence of COVID-19. Even though our sample was unbalanced, with significantly younger GPs in Zagreb and Zagreb’s County, analysing sex, age, and occupation sample was shown to be representative. The main limitation lies in the unknown prevalence of stress, anxiety, depression, and PTSD in primary HCPs in Croatia before the SARS-CoV-2 pandemic, so results obtained in this study cannot be compared with previous results. The cross-sectional design of the study limits the ability to conclude causality. There is also the consideration of possible self-report bias, which may question the authenticity of the response. Also, the study was carried out during fortnight, lacks longitudinal follow-up and was performed early in the outbreak so that results could change in the pandemic.

### Interpretation of the study results in relation to the existing literature and possible explanation of results

Our results on psychopathological outcomes of healthcare professionals are in line with previous research during SARS, MERS and with the recent SARS-CoV-2 outbreak except for our prevalence results are up to three times higher [13–15]. Reasons for the result may lie in low mental preparedness for this unique outbreak, lack of good communication between government representatives with occasional negative comments towards primary HCPs, and war experiences from 25 years ago which appear to be linked to anxiety and mood disorders [16]. Other reasons, probably typical for all HCPs globally, were social isolation, moral dilemmas, extreme workloads, and a practice environment with extensive PPE that is unfamiliar. Working in FM is characterised by a significant mental load, even without an infectious disease outbreak but now, without specificity of FPs diagnosing, mostly doing telephone or e-mail consultations, and making decisions on limited information is a new form of stress [17,18]. A survey in China found a higher risk of both anxiety and depression in physicians aged over 30, while we found a higher risk only of anxiety for older participants [19]. Female HCPs facing the COVID-19 pandemic are more vulnerable to the impact of work-related traumatic events, with higher depressive and anxiety symptoms, as confirmed in our results [20]. This evidence agrees with previous literature and a consensus that women have a two to three times higher risk of developing PTSD than men [21,22]. According to professional differences, a study conducted in the hospital reported a higher psychological burden for nurses, while our findings showed no distinction [20]. This is not surprising because the work organisation of FM practices in Croatia functions as a team with one doctor and one nurse who depend on each other. Chronic diseases and psychological disorders have a significant effect on stress, anxiety, depression, or PTSD. People with chronic diseases describe their mental health as poor, so influence on the DASS-21 and IES-R score is expected and confirmed in our outcomes [23].

Homeschooling was confirmed to be a high-stressor for parents, as found previously [24]. Reasons could range from broken routine, fear that their child will fall behind, insufficient access to technology, and impossibility to work from home and monitor their child simultaneously. Prevalence of stress, anxiety, depressive symptoms, and PTSD usually seems high after natural disasters like earthquakes, especially in women, and even years after the event [25,26]. That difference in psychological outcomes between Zagreb participants, which were hit by the quake, and other parts of Croatia had not been found. The reason for the lack of results for PTSD related to the earthquake probably lies in the timeframe after the earthquake questionnaire was conducted, since PTSD can develop even later, so we encourage further research on this subject. Also, this could be because the focus was not on the subjects who had suffered damage or grief following the quake.

Higher incidence of COVID-19 in Zagreb and Split-Dalmatia’s County means more severe cases of the disease, deaths, and supply shortages, creating a highly stressful work process, unpredictable environment, and threat for the HCPs with possible development of PTSD among our participants.

### Implications

Our results implicate that supporting mental health and well-being is as vital as securing the sustained capacity of the primary HCPs workforce. Current understanding of how to mitigate the adverse mental health impacts of this pandemic emphasises the importance of adequately preparing staff for the challenges ahead. Health policymakers and authorities should include changes to protocols that enable enhancement and coping strategies during times of increased stress. The Health Ministry’s advisory team should always have a primary care representative. To bolster staff resilience and enhance one's capacity to cope during times of increased stress, a staff support program, ‘psychological first aid’ training courses, and access to informal psychological support when needed should be implemented regularly. Since prevalence and risk factors before the pandemic were not known, long-term psychological implications of this population are worth further investigation.

## Conclusion

The high prevalence of stress, anxiety, depression, and PTSD in FM during the COVID-19 outbreak in Croatia shows a great mental load of HCPs and is associated with female sex, pre-existing chronic conditions, working in a high incidence of SARS-CoV-2 region, and having schoolchildren.
